# High Performance Drain Engineered InGaN Heterostructure Tunnel Field Effect Transistor

**DOI:** 10.3390/mi10010075

**Published:** 2019-01-21

**Authors:** Xiaoling Duan, Jincheng Zhang, Jiabo Chen, Tao Zhang, Jiaduo Zhu, Zhiyu Lin, Yue Hao

**Affiliations:** Wide Bandgap Semiconductor Technology Disciplines State Key Laboratory, School of Microelectronics, Xidian University, Xi’an 710071, China; jbchen@stu.xidian.edu.cn (J.C.); zhangtao9204@sina.com (T.Z.); jdzhu@xidian.edu.cn (J.Z.); zylin@xidian.edu.cn (Z.L.); yhao@xidian.edu.cn (Y.H.)

**Keywords:** drain engineered, tunnel field effect transistor (TFET), polarization, ambipolar, subthreshold, ON-state

## Abstract

A drain engineered InGaN heterostructure tunnel field effect transistor (TFET) is proposed and investigated by Silvaco Atlas simulation. This structure uses an additional metal on the drain region to modulate the energy band near the drain/channel interface in the drain regions, and increase the tunneling barrier for the flow of holes from the conduction band of the drain to the valence band of the channel region under negative gate bias for n-TFET, which induces the ambipolar current being reduced from 1.93 × 10^−8^ to 1.46 × 10^−11^ A/μm. In addition, polar InGaN heterostructure TFET having a polarization effect can adjust the energy band structure and achieve steep interband tunneling. The average subthreshold swing of the polar drain engineered heterostructure TFET (DE-HTFET) is reduced by 53.3% compared to that of the nonpolar DE-HTFET. Furthermore, I_ON_ increases 100% from 137 mA/mm of nonpolar DE-HTFET to 274 mA/mm of polar DE-HTFET.

## 1. Introduction

Tunnel field effect transistors (TFETs) have been considered as attractive alternative replacements to metal-oxide-semiconductor field effect transistors (MOSFETs) for low power applications [1,2,3], due to the conductive mechanism of band to band tunneling, realizing a steep subthreshold swing (SS) (less than 60 mV/dec at room temperature), good immunity against Short Channel Effects (SCEs) and high ON-state current (I_ON_) to OFF-state current (I_OFF_) ratio (I_ON_/I_OFF_) [1,4,5]. Although TFETs have various benefits, there are still some problems to be solved, such as the ambipolar behavior [6,7], low ON-state current (I_ON_) [8], and less-than-idea SS. To solve these problems, different techniques such as the use of high-k dielectric materials [9], heterojunction engineering [10,11,12,13], source pocket based devices [14,15], junction-less concept based devices [16,17,18], and narrow bandgap materials have been investigated to boost I_ON_. Drain doping profile investigation [7], gate-drain electrode gap control [19], the hetero-dielectric box concept [20], and heterojunction engineering have been developed to restrain ambipolar behavior.

Ш Nitride is the direct bandgap semiconductor, and its bandgap can be modulated from 0.7 eV (InN) to 6.2 eV (AlN), while the natural polarization effect will facilitate the formation of a steep energy band at the tunneling junction of the heterostructure, inducing a steep tunneling junction, small subthreshold swing and large ON-state current [21]. Recently, it has been reported that Ш Nitride TFETs exhibit superior device characteristics, showing the great potential of the application in the low power field [21,22,23,24]. However, the research on Ш Nitride TFET devices is just beginning, and it is worth further study.

In this paper, the drain engineering method uses workfunction engineering on an additional metal to modulate the energy band on the drain/channel interface in the drain regions and increase the tunneling barrier for the flow of holes from the conduction band of the drain to the valence band of the channel region under negative gate bias for n-TFET, which will reduce the ambipolar current of InGaN heterojunction TFETs (HTFETs). In addition, the improvement mechanism of the polarization effect on the subthreshold and ON-state characteristics of polar InGaN HTFETs are investigated at length.

The remaining paper is organized as follows: Section 2 presents the device structure, simulation models and material parameters. Section 3 is dedicated to results and discussions, including the drain engineered TFET used to suppress the ambipolar current of InGaN HTFET, and the performance comparison and analysis of polar and nonpolar HTFETs. Finally, Section 4 concludes the paper with some important findings.

## 2. Device Structure and Simulation Parameters

**Figure 1:**
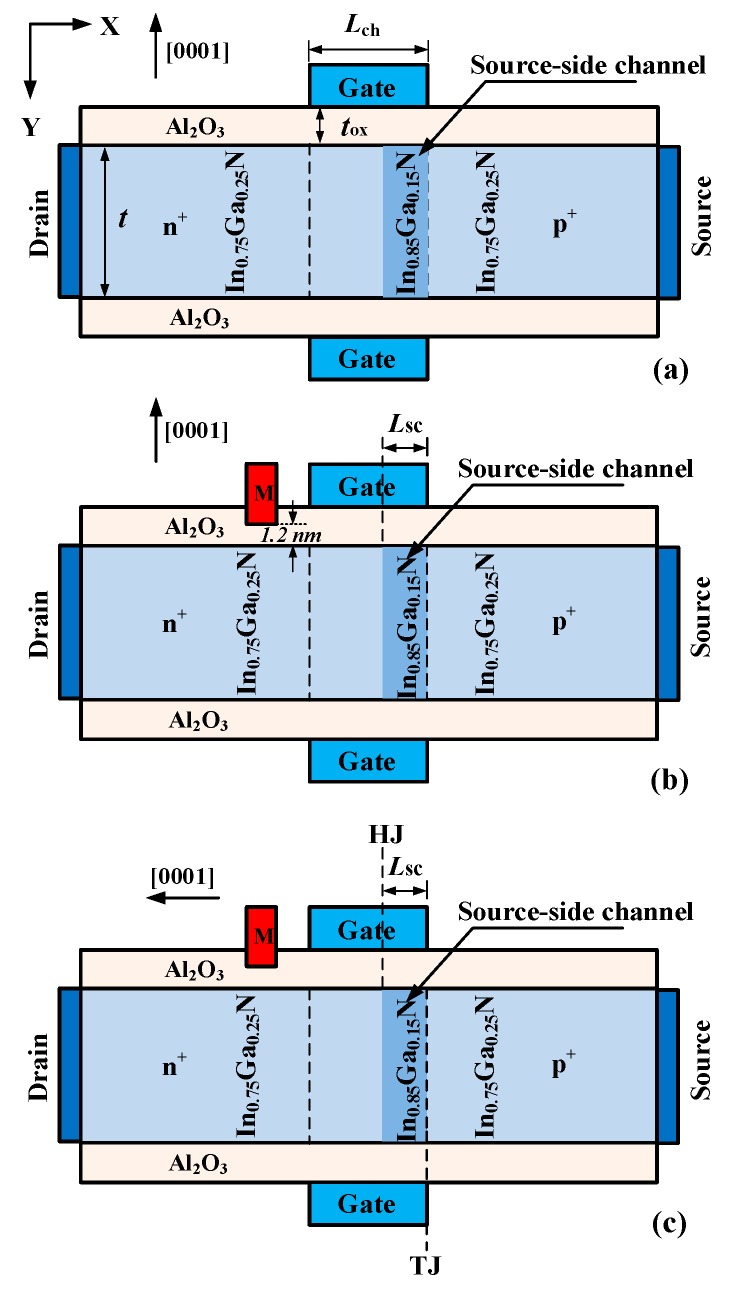
Cross-sectional views of the nonpolar (**a**) conventional, (**b**) drain engineered InGaN tunnel field effect transistor (TFET), and (**c**) polar drain engineered InGaN TFET.

Figure 1 shows the cross-sectional views of InGaN nonpolar heterostructure TFET (HTFET), InGaN nonpolar drain engineered heterostructure TFET (DE-HTFET), and InGaN polar DE-HTFET, respectively. The InGaN HTFET contains a source-side channel to improve the ON-state current and reduce the subthreshold swing. This work has been reported in reference [24]. The channel length (L_ch_) and the channel thickness (*t*) are set as 50 nm, and 10 nm, respectively. The drain metal and the gate (L_GD_) are 100 nm. The gate dielectric material is Al_2_O_3_ with a thickness (*t*_ox_) of 3 nm. The doping concentrations in the source, the channel, and the drain region are p+ 9.9 × 10^19^ cm^−3^, n 1 × 10^15^ cm^−3^, and n+ 1.2 × 10^19^ cm^−3^, respectively. The workfunction of gate metal is 5.1 eV. The proposed nonpolar DE-HTFET contains an additional drain engineering metal with the length of 10 nm and a dielectric thickness of 1.2 nm to modulate the energy band in the drain/channel region and finally reduce the ambipolar current. Along with this, the polar DE-HTFET is proposed to modulate the energy band at the source/channel tunneling junction, and will further improve the device characteristics.

Simulations are carried out using a 2D Silvaco Atlas simulator (5.19.20.R, Silvaco Inc., Santa Clara, CA, USA) [25]. The nonlocal band to-band tunneling (BTBT) is incorporated in the simulation for the calculation of the tunneling rate of charge carriers. The Shockley–Read–Hall (SRH) carrier recombination model, bandgap narrowing (BGN) model, constant low field mobility model, and field-dependent mobility model at high electric fields are activated using the SRH, BGN and FLDMOB parameters, respectively. The polarization effect in the polar TFET is simulated by a fixed polarization charge at the heterojunction interface [26], and the density of polarization surface charge (σ_pol_) will be discussed in Section 3.2. Other material parameters used in the simulations are presented in Table 1.

In the electrical characteristics analysis below, I_ON_ is defined to be the drain current I_D_ at V_G_ = V_D_ = 1 V. The average sub-threshold swing (SS_avg_) is obtained from the I_D_-V_G_ curve, it is given by

(1) $${\mathrm{SS}}_{\mathrm{avg}}{=(V}_{\mathrm{TH}}{-V}_{\mathrm{OFF}})/{(\mathrm{logI}}_{V_{\mathrm{TH}}}{-\mathrm{logI}}_{\mathrm{OFF}})$$

where the threshold voltage V_TH_ is defined to be the gate voltage (V_G_) at current of 1 × 10^−7^ A/mm, and V_OFF_ is the gate voltage at I_D_ of 10^−18^ A/mm.

## 3. Results and Discussions

### 3.1. Drain Engineered HTFET to Suppress Ambipolar Current

#### 3.1.1. Impact of Drain Engineering Metal Position on Electrical Characteristic of DE-HTFET

Drain engineered HTFET using an additional drain engineering metal (M) to modulate the energy band can increase the tunneling barrier for the flow of holes on the drain/channel interface, and reduce the ambipolar current at the negative V_G_ bias. The M position will obviously affect the electrical characteristic of DE-HTFET. The following simulations are discussed with the different M position at the M workfunction (φ_M_) of 5.15 eV.

**Figure 2:**
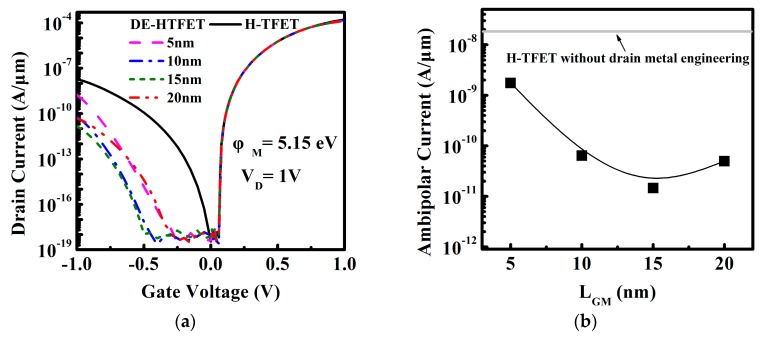
(**a**) Transfer characteristic of HTFET and DE-HTFET with varied L_GM_ at V_D_ of 1 V and φ_M_ of 5.15 eV, and (**b**) ambipolar current with different L_GM_ extracted from the I-V curve.

Figure 2 displays the I-V curves of HTFET without M and DE-HTFET with the different gate to M space (L_GM_). HTFET without drain engineering metal shows an ambipolar current of 1.93 × 10^−8^ A/μm. Overall, I_ambipolar_ of DE-HTFET by drain metal engineering has been obviously reduced compared with that of HTFET without M. As L_GM_ increases from 5 nm to 20 nm, I_ambipolar_ reduces firstly and then increases. At L_GM_ of 15 nm, DE-HTFET obtains the smallest I_ambipolar_ (1.46 × 10^−11^ A/μm), while the ON-state current (I_ON_) is almost not degraded.

**Figure 3:**
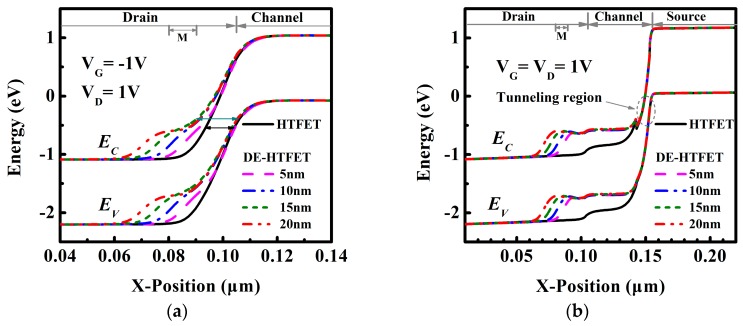
(**a**) Energy band diagram of DE-HTFET near the drain/channel tunneling junction with L_GM_ = 15 nm at V_G_ of −1 V, V_D_ of 1 V, and (**b**) Energy band diagram of DE-HTFET at ON-state.

To gain a better insight into the variation of I_ambipolar_ and I_ON_, energy band profiles for V_G_ of −1 V and 1 V are shown in Figure 3a,b, respectively. Figure 3a shows the energy band of DE-HTFET near the drain/channel junction at V_G_ of −1 V. With drain metal engineering, the tunneling distance of DE-HTFET at the negative gate bias is larger than that of HTFET. The tunneling distance of DE-HTFET firstly increases and then decreases with the increase of L_GM_. DE-HTFET with L_GM_ = 15 nm has the largest tunneling distance in the L_GM_ range from 5 nm to 20 nm. The larger tunneling distance will contribute to a lower electric field (E) and thereby a smaller tunneling rate, as illustrated with Kane’s formula [27]:

(2) $$P_{\mathrm{tun}}\sim \frac{E^{2}m_{r}^{1/2}}{E_{g}^{1/2}}\exp\left(-\frac{C_{2}m_{r}^{1/2}E_{g}^{3/2}}{E}\right),$$

where E is the electric field, C_2_ is a constant and m_r_ is effective mass. This can well explain the variation tendency of I_ambipolar_. Thus, DE-HTFET with L_GM_ of 15 nm has the smallest I_ambipolar_. Figure 3b shows the energy band of DE-HTFET at the ON-state (V_G_ = V_D_ = 1 V). It is obvious that L_GM_ variation has almost no influence over the tunneling distance at the source/channel junction, therefore I_ON_ is almost not degraded. Considering the aforementioned factors, L_GM_ of 15 nm is selected in the following discussions.

#### 3.1.2. Impact of Drain Engineering Metal Workfunction (φ_M_) on Electrical Characteristic of DE-HTFET

**Figure 4:**
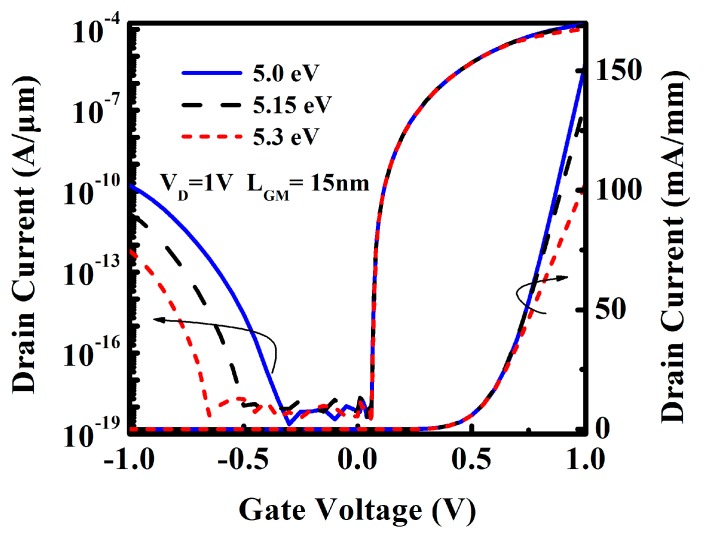
Transfer characteristic of DE-HTFET with various φ_M_ at V_D_ of 1 V.

In this section, in order to find the optimal drain engineering metal, the impacts of the various of workfunction are studied in detail. The function of the drain engineering metal is to increase the tunneling barrier of the drain/channel junction at the negative V_G_ bias, so a relatively high work function of the drain engineering metal may be a good choice. Here, electrical characteristics of DE-HTFET with drain engineering metal φ_M_ = 5.0, 5.15, and 5.3 eV are simulated, respectively, and the results are shown in Figure 4. Based on the results, it is indicated that I_ambipolar_ presents an exponential decrease trend with the increment of φ_M_, and I_ON_ stays in the same level. However, there is also a balance between I_ambipolar_ and I_ON_, that is to say, a higher workfunction is not always better. I_ON_ also reduces with the increment of φ_M_, although it is in the same level. As φ_M_ increases from 5.0 eV to 5.15 eV, I_ON_ decreases slightly. However, at φ_M_ of 5.3 eV, I_ON_ decreases significantly, which is not desirable.

**Figure 5:**
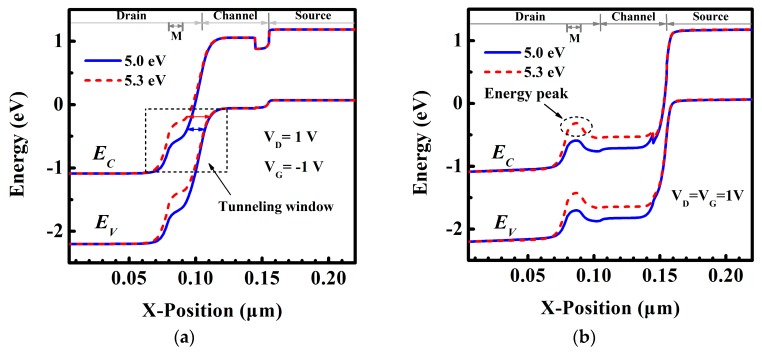
Energy band diagram at the line 1 nm away from the interface of InGaN/Al_2_O_3_ for DE-HTFET with φ_M_ = 5.0 eV and 5.3 eV as (**a**) V_D_ = 1 V, V_G_ = −1 V, and (**b**) V_D_ = V_G_ = 1 V.

To gain a better insight into the variation of I_ambipolar_ and I_ON_, Figure 5 exhibits the energy band diagram of DE-HTFET with φ_M_ = 5.0 eV and 5.3 eV for VG of −1 V and 1 V, respectively. Figure 5a shows that the tunneling distance in the tunneling window for DE-HTFET with φ_M_ = 5.3 eV is larger than that for DE-HTFET with φ_M_ = 5.0 eV, which results in the lower BTBT tunneling rate at the drain/channel interface for DE-HTFET with higher φ_M_, therefore, lower I_ambipolar_ is obtained for higher φ_M_. Figure 5b shows the energy band profile at the ON-state. An energy peak is created near the drain/channel interface, which will act as a barrier for electrons tunneling from the source to the drain region at the positive bias [28]. Therefore, I_ON_ of DE-HTFET with φ_M_ = 5.3 eV significantly degrades as shown in Figure 4. To make a trade-off between I_ambipolar_ and I_ON_, φ_M_ of 5.15 eV is selected as the last choice.

### 3.2. Polar Heterostructure DE-HTFET to Improve Device Performances

The heterostructures in TFETs studied above are all along the nonpolar plane, so no polarization charge exists at the interface of heterojunction. However, studies show that the polarization engineering in the III-nitride heterostructure can further adjust the energy band structure and achieve the interband tunneling [21]. It needs the heterostructure growing along the c-axis. Because of spontaneous polarization and piezoelectric polarization along the c-axis, large amounts of fixed polarization charge can be induced at the heterojunction interface. The polarization charge generated by polarization engineering can lead to a large internal electric field near the heterojunction interface, and increase the tunneling rate. On the other hand, polarization also changes the energy structure near the tunneling junction and helps improve TFET performance.

Herein, the polar and non-polar DE-HTFETs are compared. Figure 1c shows the schematic diagram of polar DE-HTFETs. The growth direction is along [0001], that is, the negative direction of X-axis in Figure 1. In this case, the polarization charge at the interface of III-Nitride heterojunction will be taken into account in the simulations. In particular, the polarization charge near the tunneling junction may have important effects on the device characteristics of InGaN DE-HTFET. By calculation, the density of polarization surface charge (σ_pol_) is −1.27134 × 10^13^/cm^2^ at the position of TJ, and 1.27134 × 10^13^/cm^2^ at the position of HJ. The negative value of σ_pol_ represents a negative polarization charge, and vice versa.

**Figure 6:**
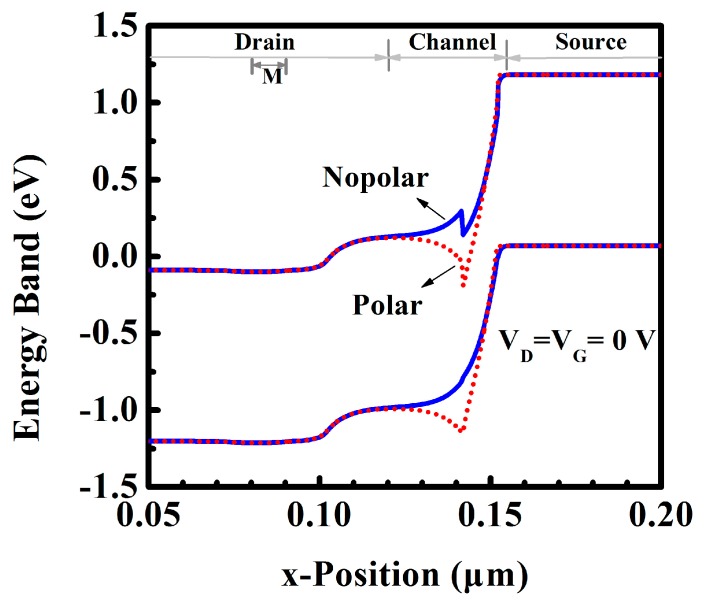
Energy band diagram in the thermal state.

**Figure 7:**
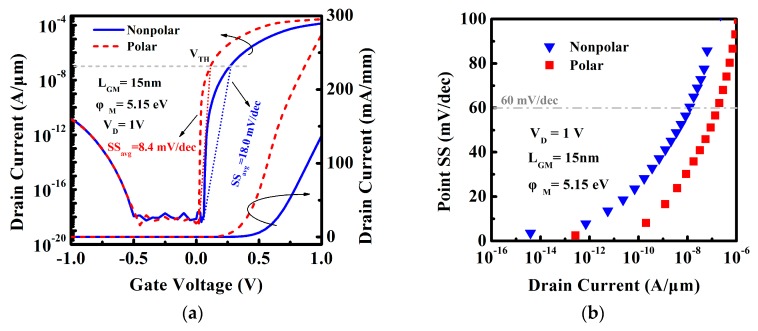
(**a**) Transfer characteristic and (**b**) point subthreshold swing for the nonpolar and polar InGaN TFET at V_D_ of 1 V.

The polarization changes the energy structure near the tunneling junction. Figure 6 shows that the polar DE-HTFET has a steeper energy band near the tunneling junction, which facilitates the better subthreshold and ON-state characteristics. Figure 7a displays the transfer characteristic of nonpolar and polar DE-HTFETs at L_GM_ of 15 nm and V_D_ of 1 V. I_D_-V_G_ curve indicates a smaller threshold voltage for the polar DE-HTFET, which will be more beneficial to the low-power applications. Besides this, the average subthreshold swing (SS_avg_) of the polar DE-HTFET is reduced by 53.3% compared to that of the nonpolar DE-HTFET. Also, I_ON_ increases 100% from 137 mA/mm of nonpolar DE-HTFET to 274 mA/mm of polar DE-HTFET. Figure 7b shows the point subthreshold swing (SS_point_) of TFETs with different I_D_ extracted from the I_D_-V_G_ curve. Obviously, SS_point_ of the polar DE-HTFET is smaller than that of the nonpolar TFET at any I_D_ value.

**Figure 8:**
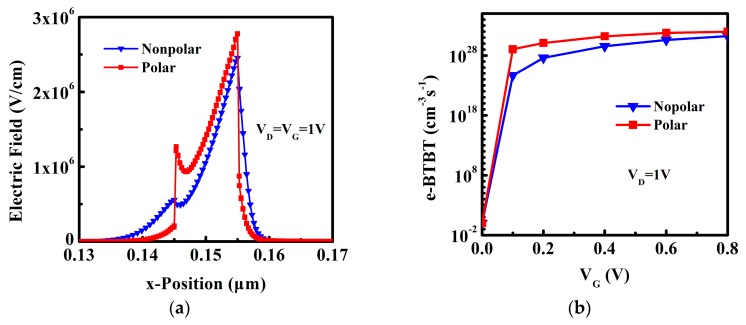
(**a**) Electric field in the ON-state, and (**b**) electron tunneling rate with various V_G._

To further investigate the mechanism of performance improvement for polar DE-HTFET, the electric field and e-BTBT rate are discussed as follows. Figure 8a exhibits that the transverse electric field intensity of the channel near the tunneling junction of polar DE-HTFET is higher than that of the non-polar device under the effect of polarization at the ON-state. Therefore, the e-BTBT rate at ON-state between the source and channel of the polar DE-HTFET is much larger than that of the nonpolar DE-HTFET as shown in Figure 7b, which is consistent with Kane’s Formula (1). This is the reason why the polar InGaN DE-HTFETs have the larger I_ON_. On the other hand, the e-BTBT rate with various of V_G_ is shown in Figure 8b. When V_G_ = 0 V, the tunneling rate of both TFETs is almost 0; when V_G_ increases to 0.1 V, the tunneling rate of polar DE-HTFET increases to 1.14 × 10^29^ cm^−3^s^−1^, however the tunneling rate of non-polar DE-HTFET is only 4.49 × 10^24^ cm^−3^s^−1^. The significant increase of the tunneling rate with the increase of V_G_ for polar DE-HTFETs in the subthreshold region makes polar DE-HTFETs have the lower subthreshold swing.

## 4. Conclusions

In summary, a drain engineered InGaN HTFET is investigated by Atlas simulation in this paper. Firstly, with the trade-off between I_ambipolar_ and I_ON_, I_ambipolar_ is reduced from 1.93 × 10^−8^ A/μm of DE-HTFET to 1.46 × 10^−11^ A/μm of HTFET by studying the impact of the drain engineering metal position and workfunction on device performances. The decreased I_ambipolar_ results from the energy band modulation by the drain engineering metal and workfunction near the drain/channel junction in the drain region. Secondly, the polarization effect induces a large internal electric field near the heterojunction interface, changes the energy structure near the tunneling junction, and helps improve TFET performance. SS_avg_ of the polar InGaN DE-HTFET is 8.4 mV/dec, which is reduced by 53.3% compared to that of the nonpolar InGaN DE-HTFET. Also, I_ON_ increases 100% from 137 mA/mm of nonpolar DE-HTFET to 274 mA/mm of polar DE-HTFET. Therefore, the structure of polar InGaN DE-HTFET embodies much more promising in low power applications.

## Figures and Tables

**Table 1:** Material parameters used in simulations [21].

Parameters	In_0.75_Ga_0.25_N	In_0.85_Ga_0.15_N
Band gap E_g_ (eV)	1.1125	0.9265
Hole effective mass (m_0_)	0.295	0.273
Electron effective mass (m_0_)	0.1025	0.0895
Static dielectric constant ε_r_	12.75	13.05
Electron mobility μ_e_ (cm^2^/Vs)	1050	1050
Hole mobility μ_h_ (cm^2^/Vs)	20	20
